# Discovery of New Eunicellin-Based Diterpenoids from a Formosan Soft Coral *Cladiella* sp.

**DOI:** 10.3390/md11114585

**Published:** 2013-11-14

**Authors:** Tsung-Hung Chen, Mei-Chin Lu, Yu-Chia Chang, Yin-Di Su, Yu-Hsin Chen, Nai-Cheng Lin, Lee-Shing Fang, Yang-Chang Wu, Ping-Jyun Sung

**Affiliations:** 1 Graduate Institute of Marine Biotechnology, National Dong Hwa University, Pingtung 944, Taiwan; E-Mails: a610162002@gmail.com (T.-H.C.); jinx6609@nmmba.gov.tw (M.-C.L.); 2 National Museum of Marine Biology and Aquarium, Pingtung 944, Taiwan; E-Mails: jay0404@gmail.com (Y.-C.C.); gobetter04@yahoo.com.tw (Y.-D.S.); kb5634@yahoo.com.tw (Y.-H.C.); lnc7222@hotmail.com (N.-C.L.); 3 Doctoral Degree Program in Marine Biotechnology, National Sun Yat-sen University and Academia Sinica, Kaohsiung 804, Taiwan; 4 Department of Marine Biotechnology and Resources and Division of Marine Biotechnology, Asia-Pacific Ocean Research Center, National Sun Yat-sen University, Kaohsiung 804, Taiwan; 5 Department of Life Science and Institute of Biotechnology, National Dong Hwa University, Hualien 974, Taiwan; 6 Department of Sport, Health and Leisure, Cheng Shiu University, Kaohsiung 833, Taiwan; E-Mail: lsfang@csu.edu.tw; 7 School of Pharmacy, College of Pharmacy, China Medical University, Taichung 404, Taiwan; E-Mail: yachwu@mail.cmu.edu.tw; 8 Chinese Medicine Research and Development Center, China Medical University Hospital, Taichung 404, Taiwan; 9 Center for Molecular Medicine, China Medical University Hospital, Taichung 404, Taiwan; 10 Graduate Institute of Natural Products, Kaohsiung Medical University, Kaohsiung 807, Taiwan

**Keywords:** eunicellin, *Cladiella*, cladieunicellin, litophynin, cytotoxicity

## Abstract

A new eunicellin diterpenoid, cladieunicellin I (**1**), and a new natural eunicellin, litophynin I diacetate (**2**), were isolated from a Formosan soft coral identified as *Cladiella* sp. The structures of eunicellins **1** and **2** were elucidated by spectroscopic methods and by comparison of the spectral data with those of related analogues. Eunicellin **1** exhibited significant cytotoxicity toward the DLD-1 human colorectal adenocarcinoma cells.

## 1. Introduction

**Figure 1:**
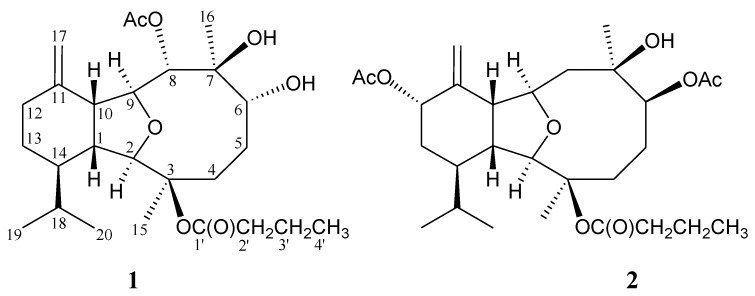
The structures of cladieunicellin I (**1**) and litophynin I diacetate (**2**).

In our continuing research on the chemical constituents of octocorals belonging to the genus *Cladiella* (family Alcyoniidae) collected off the waters of Taiwan and Indonesia, a series of interesting eunicellin-related diterpenoids (2,11-cyclized cembranoid) were isolated [1,2,3,4,5,6] and the compounds of this type were proven to possess various bioactivities [7,8,9,10,11,12,13,14,15,16,17]. Recently, our chemical examination on an octocoral identified as *Cladiella* sp. has resulted in the isolation of two eunicellin-type diterpenoids, incuding a new metabolite, cladieunicellin I (**1**) (Figure 1 and Supplementary Figures S1–S9)and a new natural eunicellin, litophynin I diacetate (**2**) [18] (Figure 1 and Supplementary Figures S10–S13). In this paper, we describe the isolation, structure determination and cytotoxicity of eunicellins **1** and **2**.

## 2. Results and Discussion

Cladieunicellin I (**1**) was isolated as a colorless oil and its molecular formula was established as C_26_H_42_O_7_ (six degrees of unsaturation) from a sodiated molecule at *m/z* 489 in the ESIMS and further supported by the HRESIMS at *m/z* 489.2825 (calcd for C_26_H_42_O_7_Na, 489.2828). The presence of hydroxy and ester groups in **1** were suggested by the IR absorptions at 3457 and 1736 cm^−1^. In the ^13^C spectrum of **1** (Table 1), two ester carbonyl resonances were identified at δ_C_ 171.5 and 173.1. One of these signals was identified as an acetate carbonyl by the presence of a methyl resonance in the ^1^H NMR spectrum at δ_H_ 2.05 (3H, s) and the other one was identified as an *n*-butyrate carbonyl by the presence of seven contiguous protons at δ_H_ 1.01 (3H, t, *J* = 7.5 Hz), 1.68 (2H, m), 2.51 (1H, dt, *J* = 16.0, 7.5 Hz) and 2.67 (1H, dt, *J* = 16.0, 7.5 Hz). From the ^13^C NMR data, an exocyclic carbon-carbon double bond was deduced from the signals at δ_C_ 110.1 (CH_2_-17) and 147.7 (C-11), and confirmed by two olefin proton signals at δ_H_ 4.58 (1H, s, H-17) and 4.73 (1H, s, H-17) in the ^1^H NMR spectrum. In addition, a suite of resonances of proton signals at δ_H_ 2.25 (1H, dd, *J* = 11.5, 7.0 Hz, H-1), 3.41 (1H, dd, *J* = 7.0, 7.0 Hz, H-10), 3.61 (1H, s, H-2) and 3.77 (1H, dd, *J* = 10.0, 7.0 Hz, H-9) and carbon signals at δ_C_ 46.1 (CH-1), 52.0 (CH-10), 93.0 (CH-2) and 78.8 (CH-9), indicated the presence of a tetrahydrofuran moiety. Comparison of the ^13^C NMR and DEPT spectra with the molecular formula indicated that there must be two exchangeable protons, requiring the presence of two hydroxy groups. From the above data, three degrees of unsaturation were accounted for and, therefore, **1** must be tricyclic.

**Table 1:** ^1^H (500 MHz, CDCl_3_) and ^13^C (125 MHz, CDCl_3_) NMR data, ^1^H–^1^H COSY and HMBC correlations for eunicellin **1**.

Position	δ_H_ (*J* in Hz)	δ_C_, Multiple	^1^H–^1^H COSY	HMBC
1	2.25 dd (11.5, 7.0)	46.1, CH	H-10, H-14	C-9, -10, -14, -18
2	3.61 s	93.0, CH	n.o.	C-1, -3, -9, -10, -14, -15
3		85.2, C		
4	2.50 m; 1.97 m	29.2, CH_2_	H_2_-5	C-2, -3, -6, -15
5	2.08 m; 1.70 m	23.6, CH_2_	H_2_-4, H-6	C-3
6	3.69 dd (12.5, 11.0)	76.3, CH	H_2_-5, OH-6	C-4, -7, -16
7		78.6, C		
8	5.40 d (10.0)	79.2, CH	H-9	C-7, -9, -10, -16, acetate carbonyl
9	3.77 dd (10.0, 7.0)	78.8, CH	H-8, H-10	C-2, -7, -8, -11
10	3.41 dd (7.0, 7.0)	52.0, CH	H-1, H-9	C-1, -8, -9, -11, -12, -14, -17
11		147.7, C		
12	2.30 br d (12.5); 2.01 m	31.6, CH_2_	H_2_-13	n.o.
13	1.78 m; 1.06 m	25.2, CH_2_	H_2_-12, H-14	n.o.
14	1.23 m	44.3, CH	H-1, H_2_-13, H-18	C-18
15	1.42 s	23.3, CH_3_		C-2, -3, -4
16	1.31 s	19.6, CH_3_		C-6, -7, -8
17	4.73 s; 4.58 s	110.1, CH_2_		C-10, -11, -12
18	1.70 m	29.0, CH	H-14, H_3_-19, H_3_-20	C-1, -13, -14, -19, -20
19	0.99 d (7.0)	21.9, CH_3_	H-18	C-14, -18, -20
20	0.79 d (6.5)	15.3, CH_3_	H-18	C-14, -18, -19
3-OCOCH_2_CH_2_CH_3_				
1′		173.1, C		
2′	2.67 dt (16.0, 7.5); 2.51 dt (16.0, 7.5)	36.7, CH_2_	H_2_-3	C-1′, -3′, -4′
		
3′	1.68 m	18.5, CH_2_	H_2_-2′, H_3_-4′	C-1′, -2′, -4′
4′	1.01 t (7.5)	13.5, CH_3_	H_2_-3′	C-2′, -3′
8-OAc		171.5, C		
	2.05 s	21.4, CH_3_		Acetate carbonyl
6-OH	4.41 br d (11.0)		H-6	n.o.

n.o. = not observed.

From the ^1^H–^1^H COSY spectrum of **1** (Table 1), the separate spin systems of H_2_-4/H_2_-5/H-6, H-8/H-9/H-10/H-1 and OH-6/H-6 were differentiated. These data, together with the HMBC correlations between H-1/C-9, -10; H-2/C-1, -3, -9, -10; H_2_-4/C-2, -3, -6; H_2_-5/C-3; H-6/C-4, -7; H-8/C-7, -9, -10; H-9/C-2, -7, -8; and H-10/C-1, -8, -9, established the connectivity from C-1 to C-10 in the ten-membered ring. The 1-isopropyl-4-methylenecyclohexane ring, which is fused to the ten-membered ring at C-1 and C-10, was elucidated by the ^1^H–^1^H COSY correlations between H-1/H-14/H_2_-13/H_2_-12 and H-14/H-18/H_3_-19 (H_3_-20) and by the HMBC correlations between H-1/C-14, -18; H-2/C-14; H-9/C-11; H-10/C-11, -12, -14, -17; H-18/C-1; and H_2_-17/C-10. The isopropyl group was positioned at C-14 from the HMBC correlations between H-1/C-14, -18; H-14/C-18; H_3_-19/C-14, -18, -20; and H_3_-20/C-14, -18, -19. An exocyclic carbon-carbon double bond at C-11 was confirmed by the HMBC correlations between H_2_-17/C-10, -11, -12. The ether bridge between C-2 and C-9 was supported by the HMBC correlations between H-2/C-9 and H-9/C-2. The hydroxy proton signal at δ_H_ 4.41 was revealed by its ^1^H–^1^H COSY correlation to δ_H_ 3.69 (H-6), indicating its attachment to C-6. The location of an acetate group in **1** was confirmed by an HMBC correlation between H-8 (δ_H_ 5.40) and the acetate carbonyl (δ_C_ 171.5). Thus, the remaining *n*-butyrate and hydroxy groups were at C-3 and C-7, oxygenated quaternary carbons which bonded to the C-15 and C-16 tertiary methyls and were confirmed by the HMBC correlations between H_3_-15/C-2, -3, -4 and H_3_-16/C-6, -7, -8 and by the key characteristic ^13^C NMR signals for C-3 (δ_C_ 85.2) and C-7 (δ_C_ 78.6), respectively.

**Figure 2:**
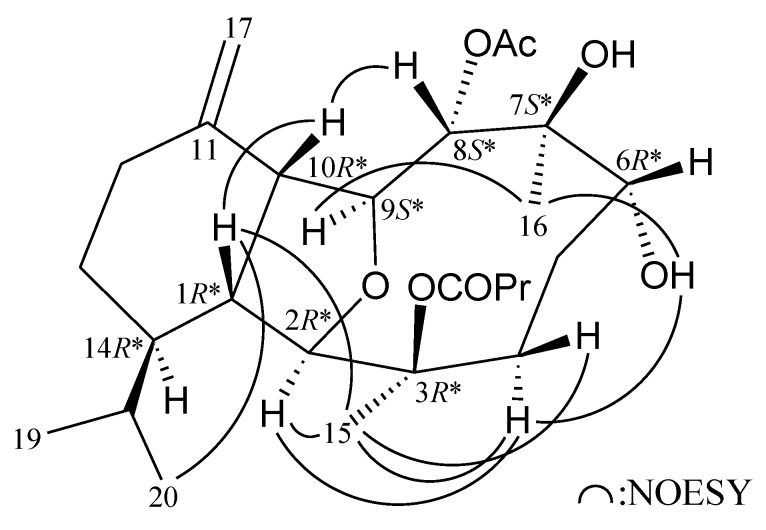
Selective NOESY correlations for **1**.

Most naturally occurring eunicellin analogues from soft corals belonging to the genus *Cladiella* have H-1 and H-10 in the β-orientation [10]. The relative configuration of **1** was elucidated mainly from a NOESY spectrum (Figure 2) and analysis of vicinal proton coupling constants analysis. In the NOESY experiment for **1**, H-1 correlated with H-10 and H_3_-20, suggesting that H-1, H-10 and the isopropyl group are situated on the same face as β protons. No coupling constant was detected between H-1 and H-2, and there was no correlation between these two protons in the NOESY experiment, indicating that the dihedral angle between H-1 and H-2 is approximately 90° and H-2 should be α-oriented. By the same token, coupling constant detected between H-8/H-9 (*J* = 10.0 Hz) and H-9/H-10 (*J* = 7.0 Hz), and there is a correlation was found between H-8 and H-10, suggesting that H-8 and H-9 were β- and α-oriented, respectively, in **1**. It was found that one of the methylene protons at C-4 (δ_H_ 1.97) exhibited a correlation with H-2, and therefore it was assigned as H-4α, and the other C-4 proton (δ_H_ 2.50) as H-4β. The correlation between H-4α and OH-6 (δ_H_ 4.41), indicating that the hydroxy group at C-6 was α-oriented. The C-15 methyl showed correlations with H-1, H-2 and H-4α/β, but not with H-10, demonstrating the *n*-butyrate group at C-3 was β-oriented. H_3_-16 showed correlations with OH-6 and H-9, suggesting the β-orientation of hydroxy group at C-7. Based on the above findings, the structure of **1** was elucidated and the chiral carbons for **1** were assigned as 1R*, 2R*, 3R*, 6R*, 7S*, 8S*, 9S*, 10R* and 14R*.

The present study also led to the isolation of a new natural eunicellin **2** [18]. Eunicellin **2** has the molecular formula C_28_H_44_O_8_ as determined by the HRESIMS at *m/z* 531.2931 (calcd for C_28_H_44_O_8_Na, 531.2934). The IR spectrum of **2** showed bands at 3413 and 1732 cm^−^^1^, consistent with the presence of hydroxy and ester groups. It was found that the ^1^H and ^13^C NMR data of **2** are identical to those of a known semi-synthetic compound, litophynin I diacetate [18]. However, eunicellin **2** has not been isolated previously from any natural sources.

Cytotoxicity of compounds **1** and **2** toward HL-60 (human promyelocytic leukemia), K562 (human erythromyeloblastoid leukemia), DLD-1 (human colorectal adenocarcinoma), HTC-116 (human colorectal carcinoma) and T-47D (human breast ductal carcinoma) showed that cladieunicellin I (**1**) exhibited selective cytotoxicity towards DLD-1 tumor cells (Table 2).

**Table 2:** Cytotoxic data of compounds **1** and **2**.

	Cell lines IC_50_ (µM)				
Compounds	HL-60	K562	DLD-1	HCT-116	T-47D
**1**	32.15	NA	1.59	NA	NA
**2**	34.21	NA	37.95	NA	NA
Doxorubicin ^a^	0.002	1.29	10.98	0.81	1.71

^a^ Doxorubicin was used as a positive control; NA = not active at 40 µM for 72 h.

## 3. Experimental Section

### 3.1. General Experimental Procedures

Optical rotations were measured on a Jasco P-1010 digital polarimeter (Japan Spectroscopic Corporation, Tokyo, Japan). Infrared spectra were recorded on a Varian Diglab FTS 1000 FT-IR spectrometer (Varian Inc., Palo Alto, CA, USA); peaks are reported in cm^−1^. NMR spectra were recorded on a Varian Inova 500 spectrometer or a Varian Mercury Plus 400 NMR spectrometer (Varian Inc.) using the residual CHCl_3_ signal (δ_H_ 7.26 ppm) as the internal standard for ^1^H NMR and CDCl_3_ (δ_C_ 77.1 ppm) for ^13^C NMR. Coupling constants (*J*) are given in Hz. ESIMS and HRESIMS were recorded using a Bruker APEX II FT mass spectrometer (Bruker, Bremen, Germany). Column chromatography was performed on silica gel (230–400 mesh, Merck, Darmstadt, Germany). TLC was carried out on precoated Kieselgel 60 F_254_ (0.25 mm, Merck); spots were visualized by spraying with 10% H_2_SO_4_ solution followed by heating. The normal phase HPLC (NP-HPLC) was performed using a system comprised of a Hitachi L-7110 pump (Hitachi Ltd., Tokyo, Japan) and a Rheodyne 7725 injection port (Rheodyne LLC, Rohnert Park, CA, USA). Two normal phase columns (Supelco Ascentis^®^ Si Cat #: 581515-U, 25 cm × 21.2 mm, 5 µm; 581514-U, 25 cm × 10 mm, 5 µm, Sigma-Aldrich. Com., St. Louis, MO, USA) were used for NP-HPLC. The reverse phase HPLC (RP-HPLC) was performed using a system comprised of a Hitachi L-7100 pump (Hitachi Ltd.), a Hitachi L-2455 photodiode array detector (Hitachi Ltd.), a Rheodyne 7725 injection port (Rheodyne LLC) and a Varian Polaris C-18-A column (250 mm × 10 mm, 5 µm; Varian Inc.).

### 3.2. Animal Material

Specimens of the octocoral *Cladiella* sp. [19] were collected by hand using SCUBA equipment off the coast of Penghu Archipelago, Taiwan in September, 2011, and stored at −20 °C until extraction. A voucher specimen (NMMBA-TWSC-11011) was deposited in the National Museum of Marine Biology and Aquarium, Taiwan.

### 3.3. Extraction and Isolation

Specimens of the soft coral *Cladiella* sp. (wet weight 1.25 kg, dry weight 457 g) were minced and extracted with ethyl acetate (EtOAc). The EtOAc extract left after removal of the solvent (12.4 g) was separated by silica gel and eluted using *n*-hexane/EtOAc in a stepwise fashion from 100:1–pure EtOAc to yield 16 fractions A–P. Fraction N (786 mg) was chromatographed on silica gel, using a mixture of *n*-hexane and acetone in a stepwise fashion from 6:1–pure acetone to obtain 15 subfractions N1–N15. Fraction N3 (96.4 mg) was repurified by NP-HPLC, using a mixture of *n*-hexane and acetone (4:1, flow rate: 2.0 mL/min) to yield five subfractions N3A–N3E. Fraction N3C (25.3 mg) was further separated by NPLC, using a mixture of *n*-hexane and acetone (4:1) to yield cladieunicellin I (**1**) (0.7 mg, *t*_R_ = 35 m). The residue of fraction N4 (153 mg) was separated by NP-HPLC, using a mixture of *n*-hexane and acetone (4:1) to obtain 7 subfractions N4A–N4G. Fraction N4E (19.6 mg) was repurified by RP-HPLC, using a mixture of methanol and water (8:2) to yield 14 fractions N4E1–N4E14. Fraction N4E11 (2.2 mg) was chromatopraphed by NP-HPLC, using a mixture of *n*-hexane and acetone (7:2, flow rate 1.0 mL/min) to yield litophynin I diacetate (**2**) (0.8 mg, *t*_R_ = 30 m).

Cladieunicellin I (**1**): colorless oil; 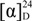 −8 (*c* 0.04, CHCl_3_); IR (neat) ν_max_ 3457, 1736 cm^−1^; ^1^H (500 MHz, CDCl_3_) and ^13^C (125 MHz, CDCl_3_) NMR data, see Table 1; ESIMS: *m/z* 489 (M + Na)^+^; HRESIMS: *m/z* 489.2825 (calcd for C_26_H_42_O_7_Na, 489.2828).

Litophynin I diacetate (**2**): colorless oil; 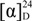 +2 (*c* 0.08, CHCl_3_); IR (neat) ν_max_ 3413, 1732 cm^−1^; ^1^H (400 MHz, CDCl_3_) δ_H_ 5.63 (1H, d, *J* = 5.6 Hz, H-6), 5.49 (1H, dd, *J* = 3.2, 3.2 Hz, H-12), 5.15 (1H, d, *J* = 1.2 Hz, H-17), 4.94 (1H, d, *J* = 1.2 Hz, H-17), 4.37 (1H, ddd, *J* = 8.4, 7.2, 7.2 Hz, H-9), 3.72 (1H, s, H-2), 3.03 (1H, dd, *J* = 7.2, 7.2 Hz, H-10), 2.61 (1H, dd, *J* = 14.4, 8.4 Hz, H-4), 2.39–2.27 (2H, m, H_2_-2′), 2.22 (1H, dd, *J* = 13.6, 7.2 Hz, H-1), 2.08 (3H, s, acetate methyl), 2.05 (3H, s, acetate methyl), 2.01 (1H, m, H-4), 1.94 (1H, ddd, *J* = 14.0, 3.6, 3.2 Hz, H-13), 1.85 (2H, m, H_2_-8), 1.81 (1H, m, H-18), 1.69 (1H, m, H-14), 1.67 (2H, m, H_2_-3′), 1.53–1.45 (2H, m, H_2_-5), 1.41 (3H, s, H_3_-15), 1.30 (1H, ddd, *J* = 14.0, 13.6, 3.2 Hz, H-13), 1.19 (3H, s, H_3_-16), 0.99 (3H, t, *J* = 7.2 Hz, H_3_-4′), 0.95 (3H, d, *J* = 6.8 Hz, H_3_-19), 0.79 (3H, d, *J* = 6.8 Hz, H_3_-20); ^13^C (100 MHz, CDCl_3_) δ_C_ 172.2 (C-1′, *n*-butyrate carbonyl), 171.8 (acetate carbonyl), 170.4 (acetate carbonyl), 142.8 (C-11), 116.8 (CH_2_-17), 91.3 (CH-2), 86.5 (C-3), 79.2 (CH-9), 84.4 (CH-6), 75.5 (C-7), 72.8 (CH-12), 51.9 (CH-10), 46.1 (CH_2_-8), 44.7 (CH-1), 37.4 (CH_2_-2′), 36.3 (CH-14), 35.6 (CH_2_-4), 29.1 (CH_2_-5), 28.5 (CH_2_-13), 28.5 (CH-18), 23.7 (CH_3_-16), 23.1 (CH_3_-15), 21.7 (CH_3_-19), 21.6 (acetate methyl), 21.4 (acetate methyl), 18.4 (CH_2_-3′), 15.3 (CH_3_-20), 13.7 (CH_3_-4′); ESIMS: *m/z* 531 (M + Na)^+^; HRESIMS: *m/z* 531.2931 (calcd for C_28_H_44_O_8_Na, 531.2934).

### 3.4. Cytotoxicity Testing

Cytotoxicity of compounds **1** and **2** was assayed with a modification of the MTT [3-(4,5-dimethylthiazol-2-yl)-2,5-diphenyltetrazolium bromide] colorimetric method according to previously described procedures [20,21].

## 4. Conclusions

A new eunicellin-based diterpenoid, cladieunicellin I (**1**) along with a new natural eunicellin, litophynin I diacetate (**2**), were isolated from the soft coral *Cladiella* sp. It is interesting to note that eunicellin **1** is much more effective against human colorectal adenocarcinoma DLD-1 tumor cells than that of positive control (doxorubicin), but this compound is not active toward another human colorectal carcinoma, HCT-116. In a previous study, a cladieunicellin analogue, cladieunicellin B, was also found to exhibit cytotoxicity toward DLD-1 tumor cells (IC_50_ = 5.95 µM) [2]. Thus, eunicellin **1** could be a promising bioactive agent and may warrant further biomedical investigation. Because octocorals are claimed to be endangered species and based on the potential medicinal use, the soft coral *Cladiella* sp. will be transplanted to culturing tanks located in the National Museum of Marine Biology and Aquarium, Taiwan, for exhibition and the extraction of additional natural products to establish a stable supply of bioactive material.

## Supplementary Files

Supplementary File 1 Supplementary Materials (PDF, 339 KB)
