# Supplementary material for: Discovery of New Eunicellin-Based Diterpenoids from a Formosan Soft Coral Cladiella sp
Source: Mar Drugs. 2013 Nov 14;11(11):4585–93. doi: 10.3390/md11114585 (PMC3853747; doi:10.3390/md11114585)
Supplement: Supplementary File 1 — Supplementary Materials (PDF, 339 KB) [file marinedrugs-11-04585-s001.pdf]

Supplementary Materials

Figure S1. ^1H NMR spectrum (500 MHz) of compound **1** in $CDCl_3$.

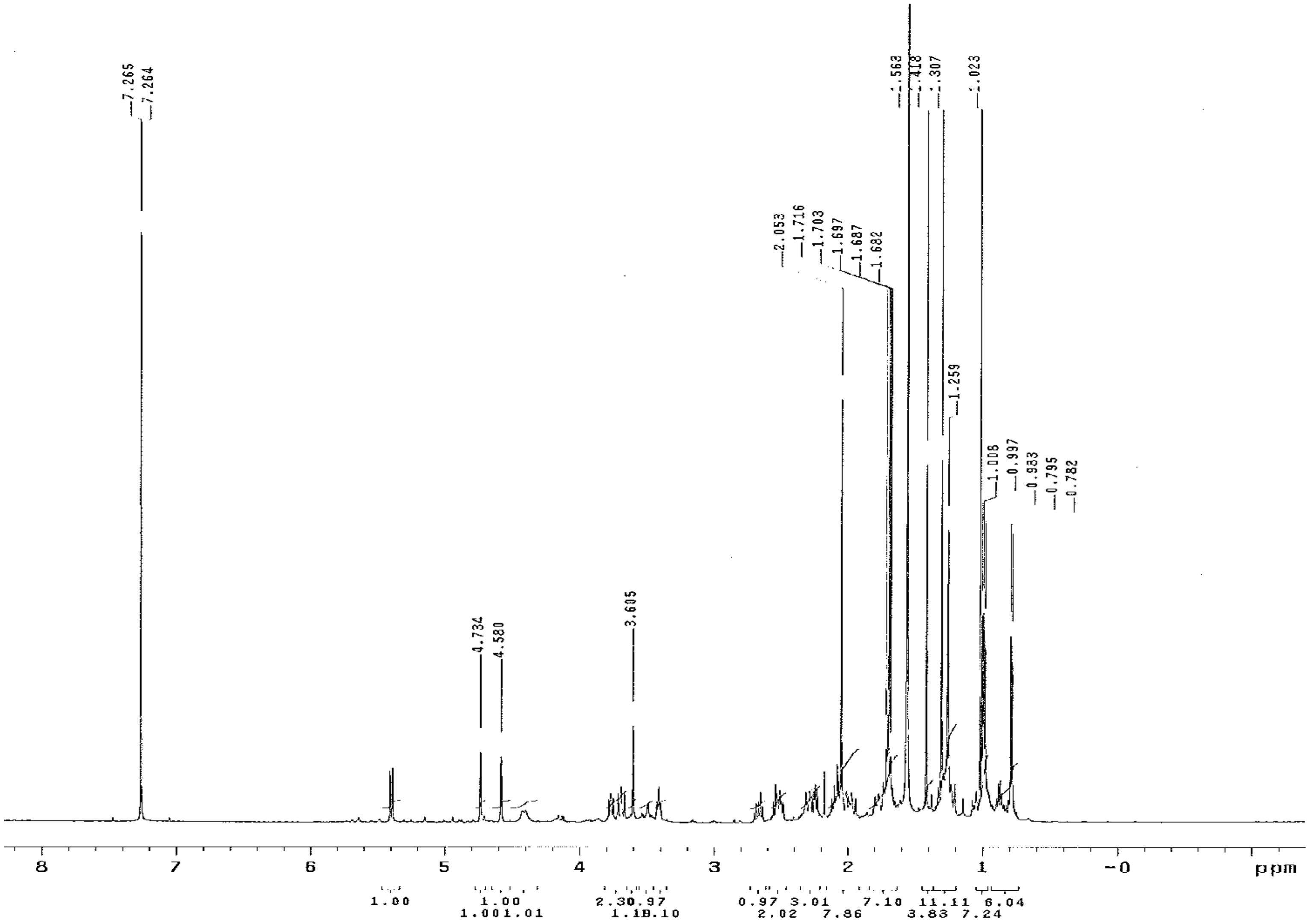

Figure S2. ^{13}C NMR spectrum (125 MHz) of compound **1** in $CDCl_3$.

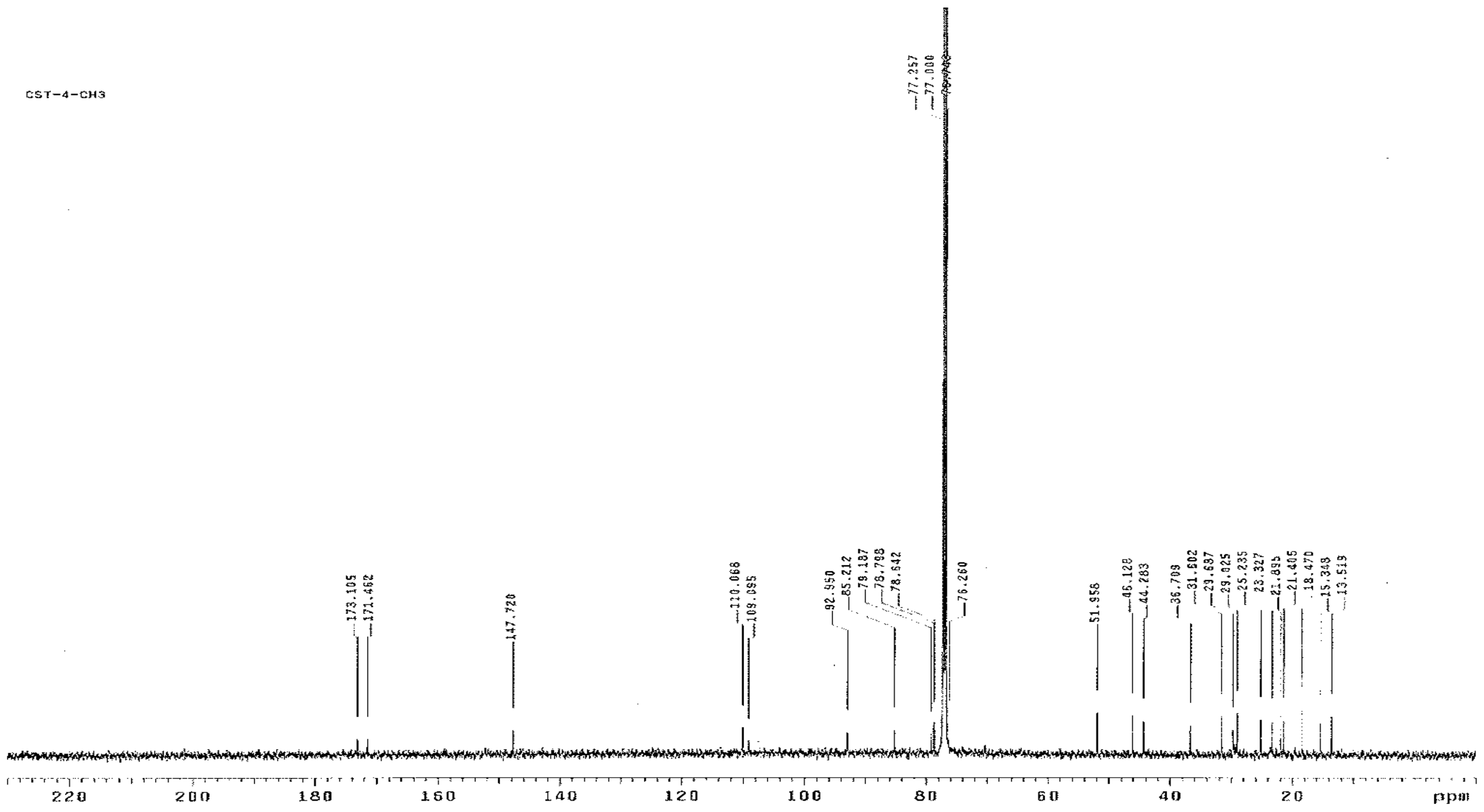

Figure S3. DEPT spectrum (125 MHz) of compound **1** in $CDCl_3$.

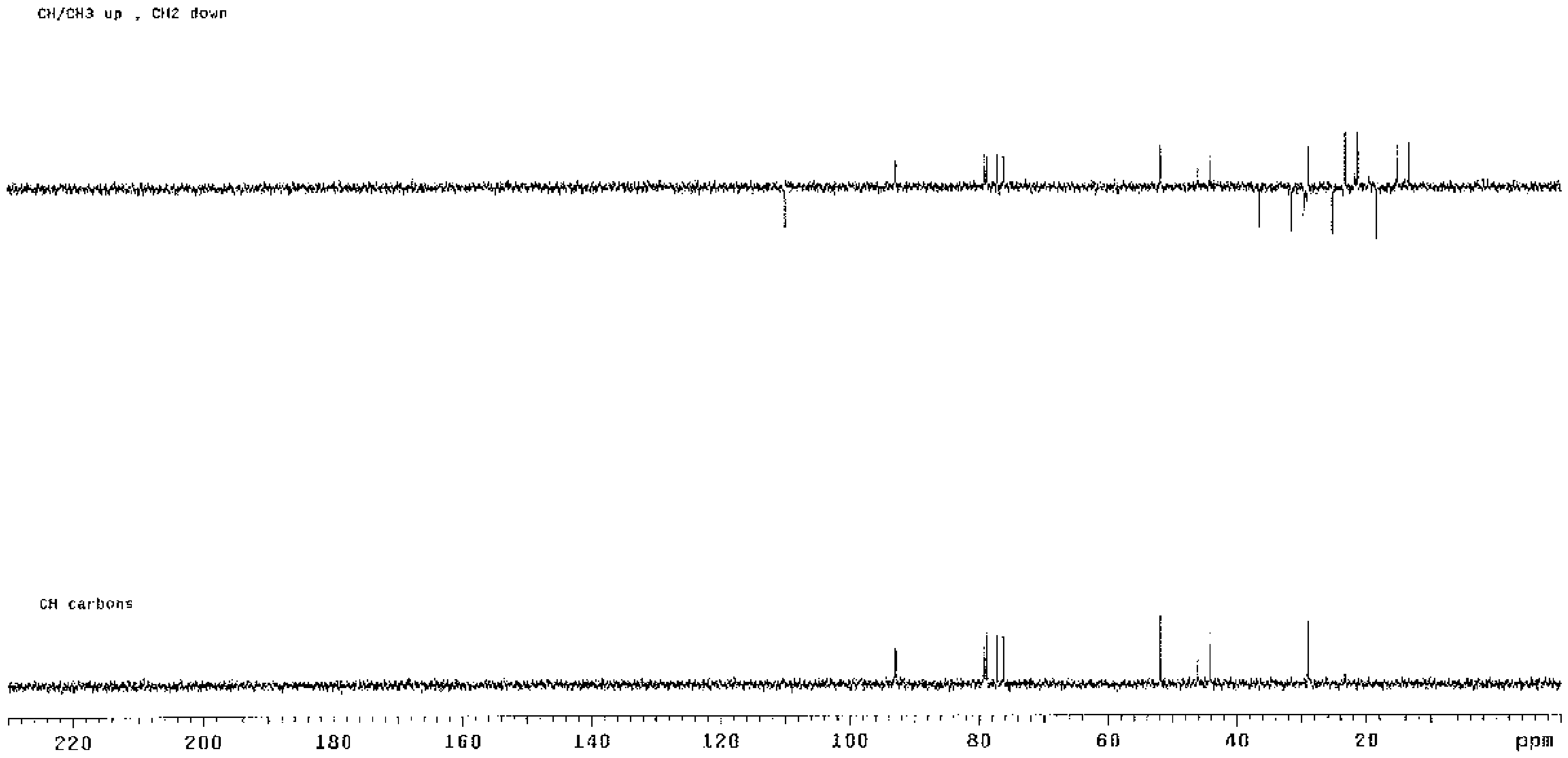

Figure S4. HSQC spectrum (500 MHz) of compound **1** in $CDCl_3$.

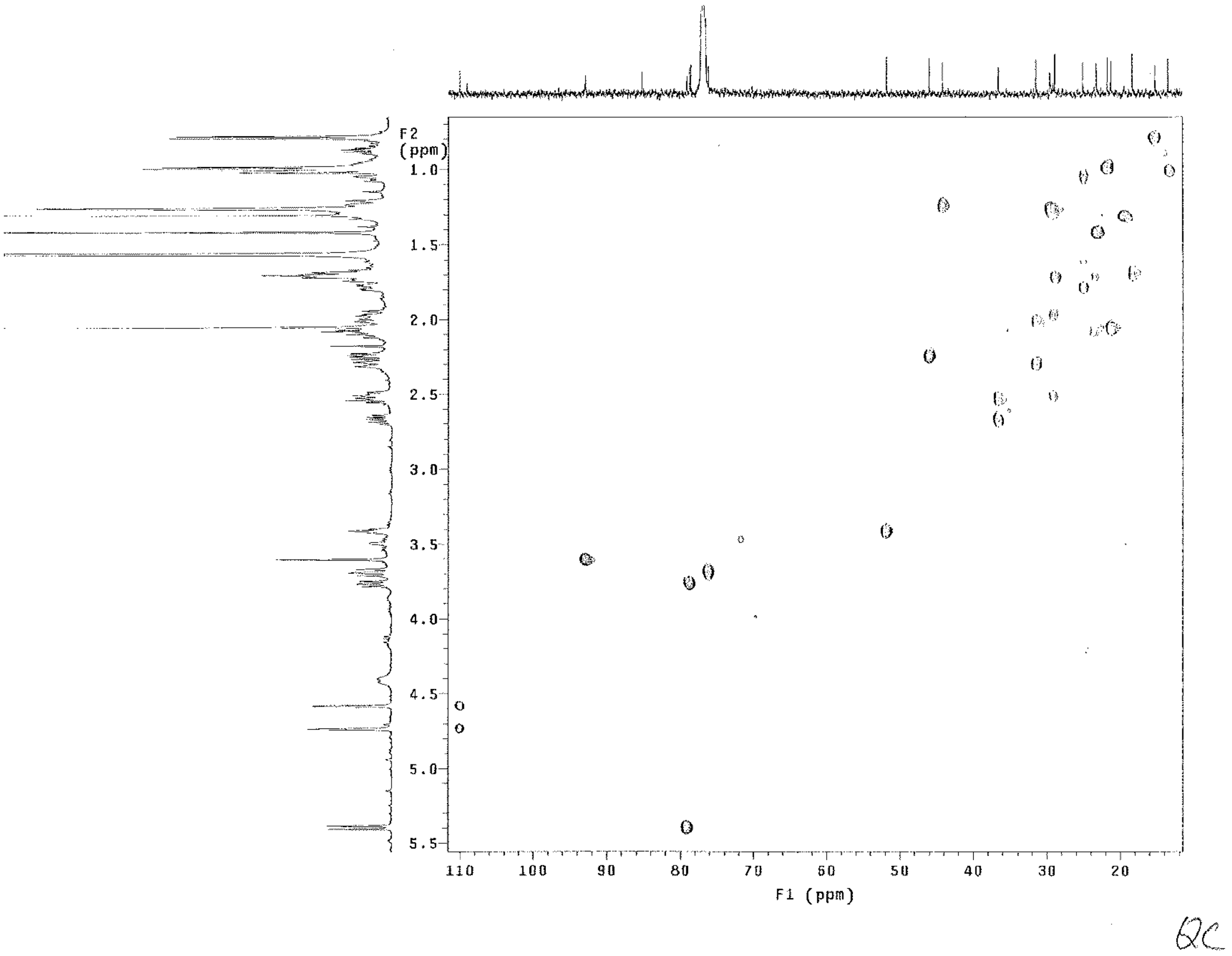

Figure S5. HMBC spectrum (500 MHz) of compound **1** in $CDCl_3$.

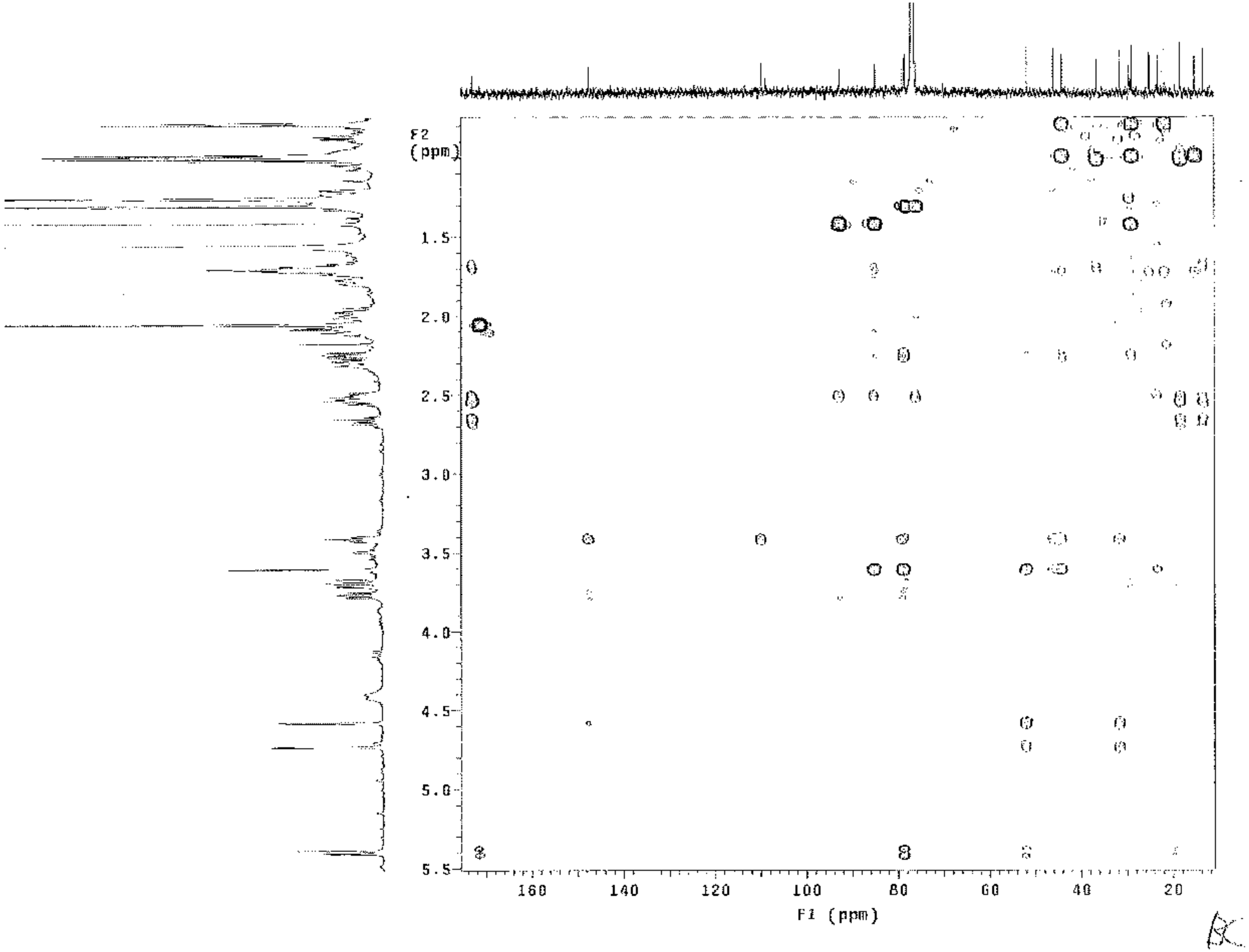

Figure S6. COSY spectrum (500 MHz) of compound **1** in $CDCl_3$.

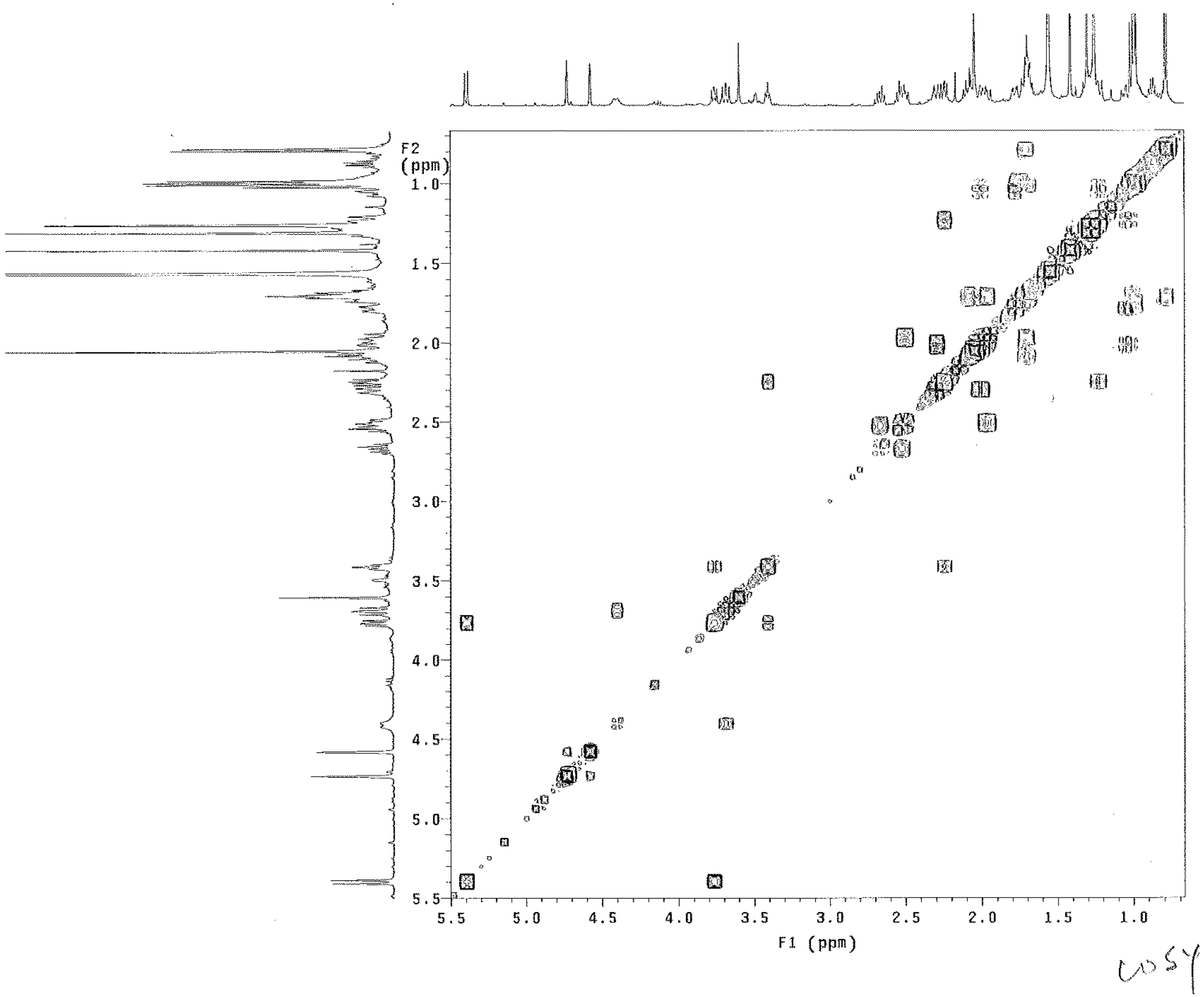

Figure S7. NOESY spectrum (500 MHz) of compound **1** in $CDCl_3$.

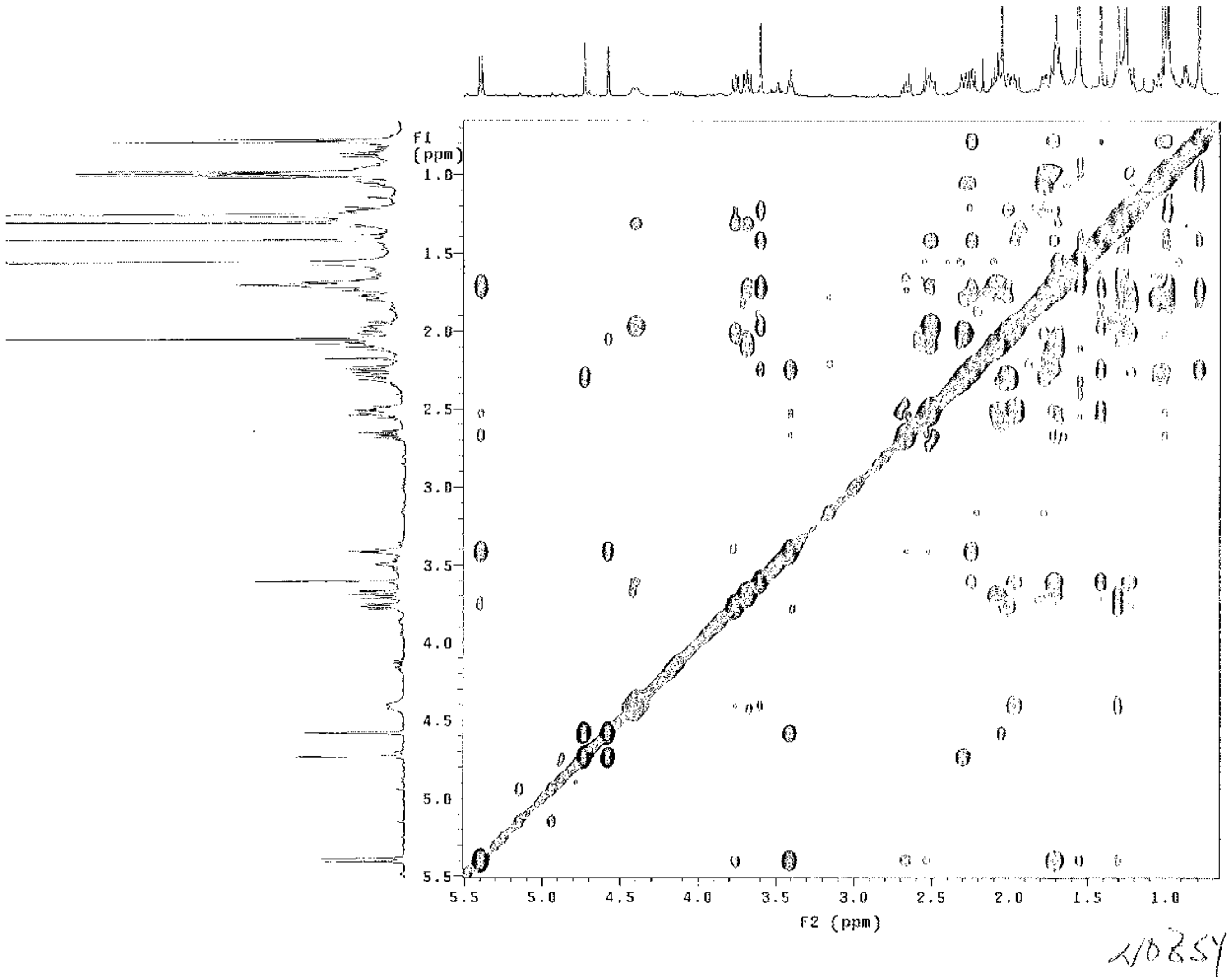

Figure S8. LRESIMS spectrum of compound **1**.

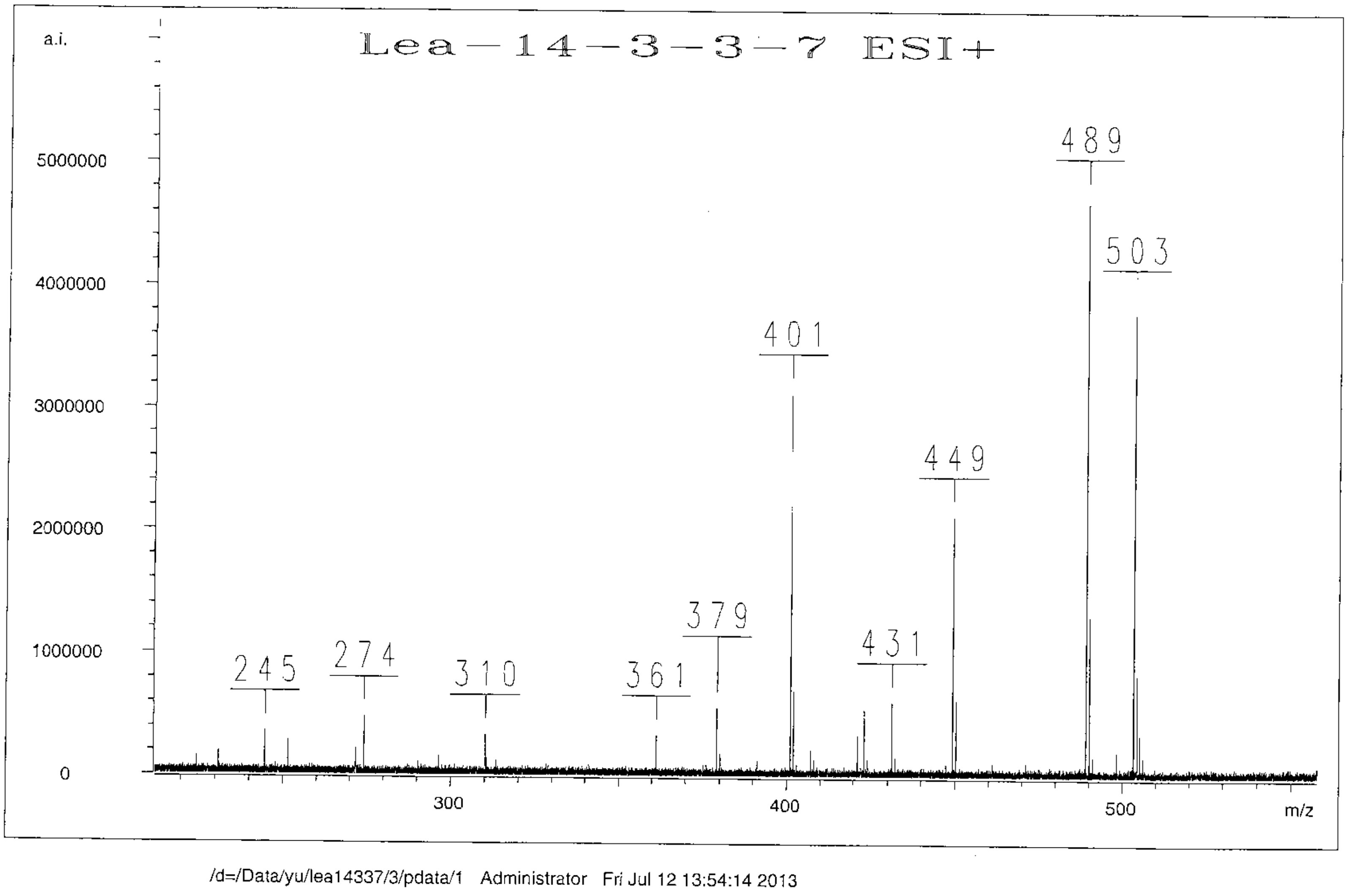

Figure S9. HRESIMS spectrum of compound **1**.

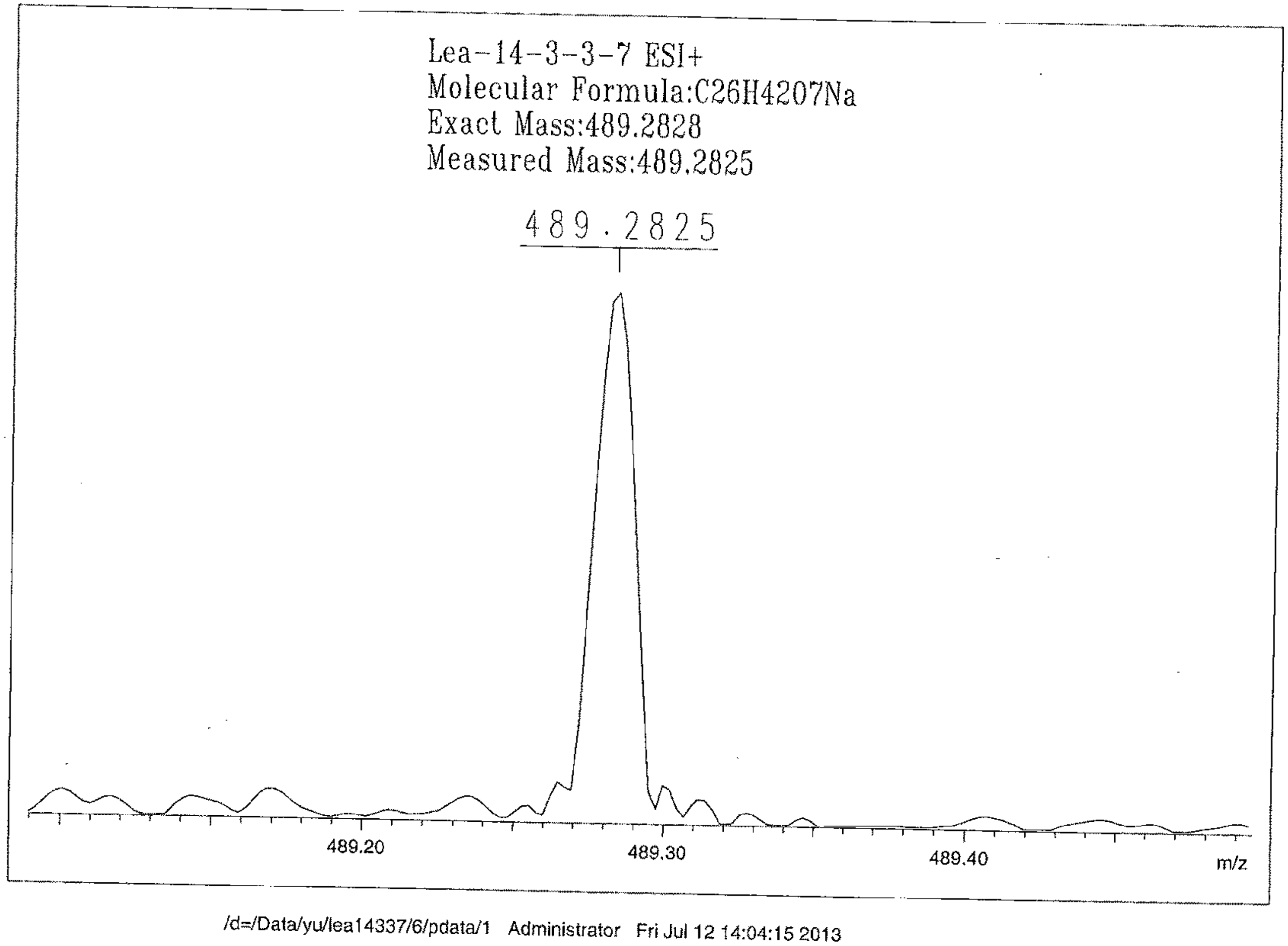

Figure S10. ^{1}H NMR spectrum (400 MHz) of compound **2** in $CDCl_3$.

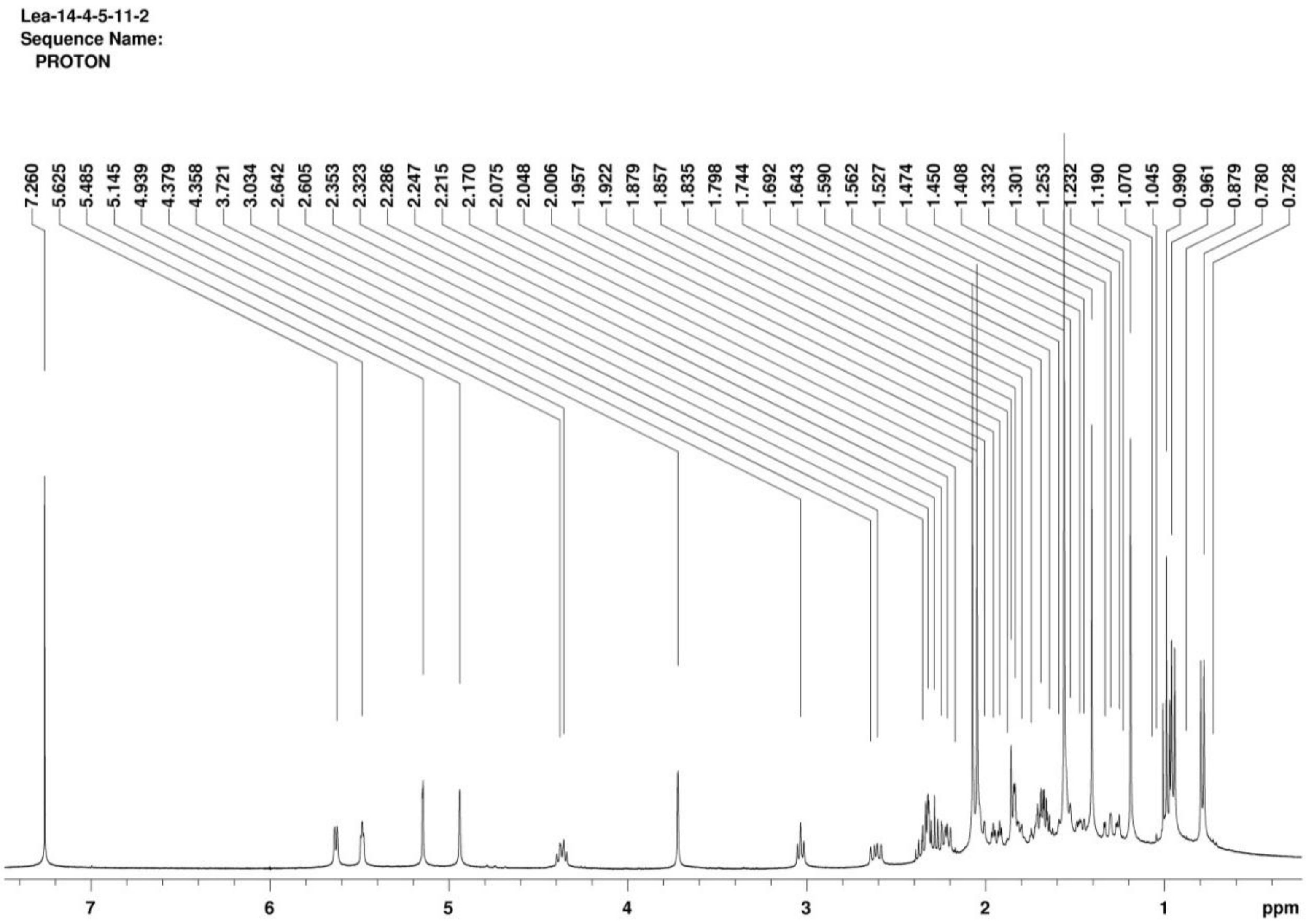

Figure S11. ^{13}C NMR spectrum (100 MHz) of compound **2** in $CDCl_3$.

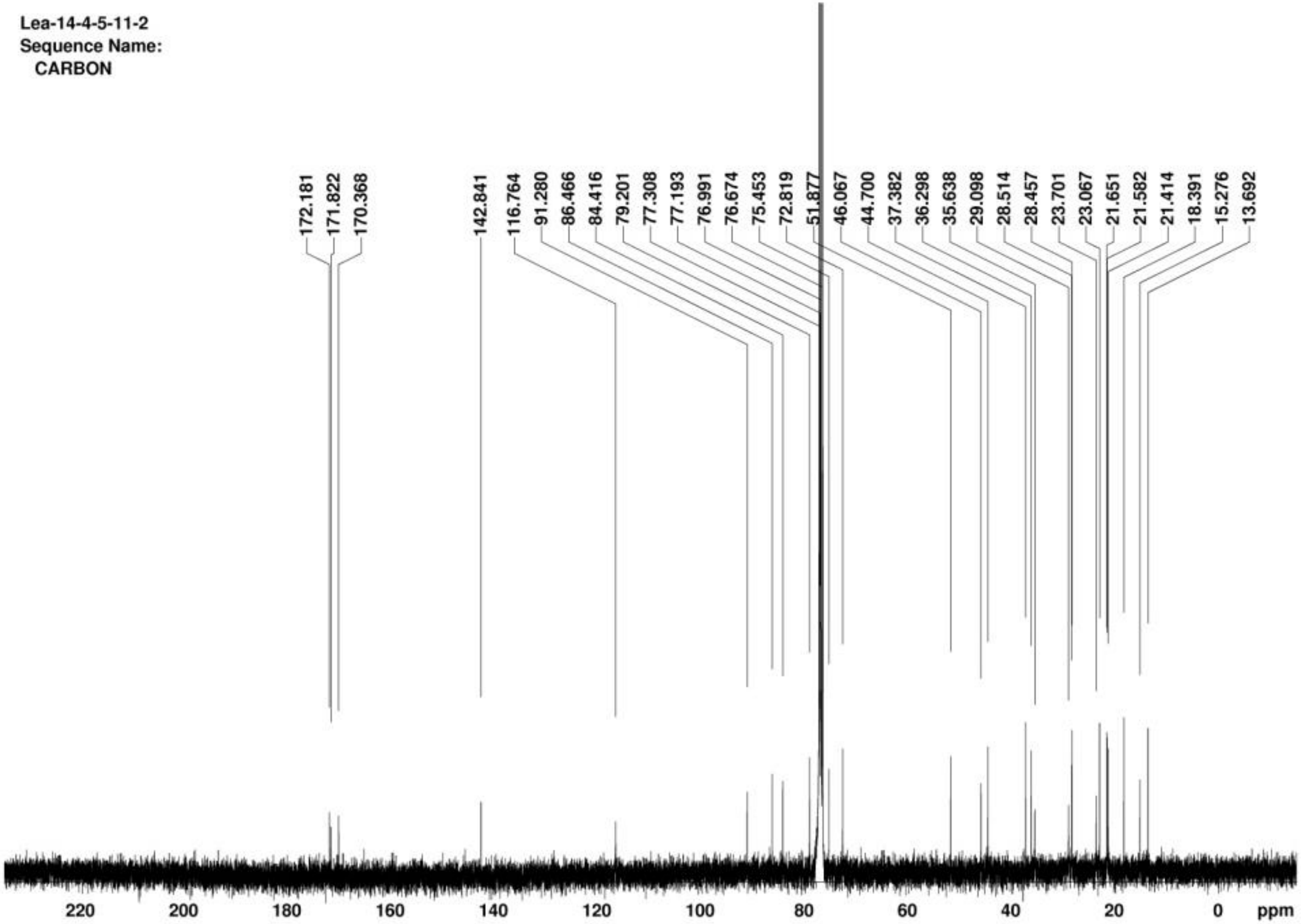

Figure S12. LRESIMS spectrum of compound **2**.

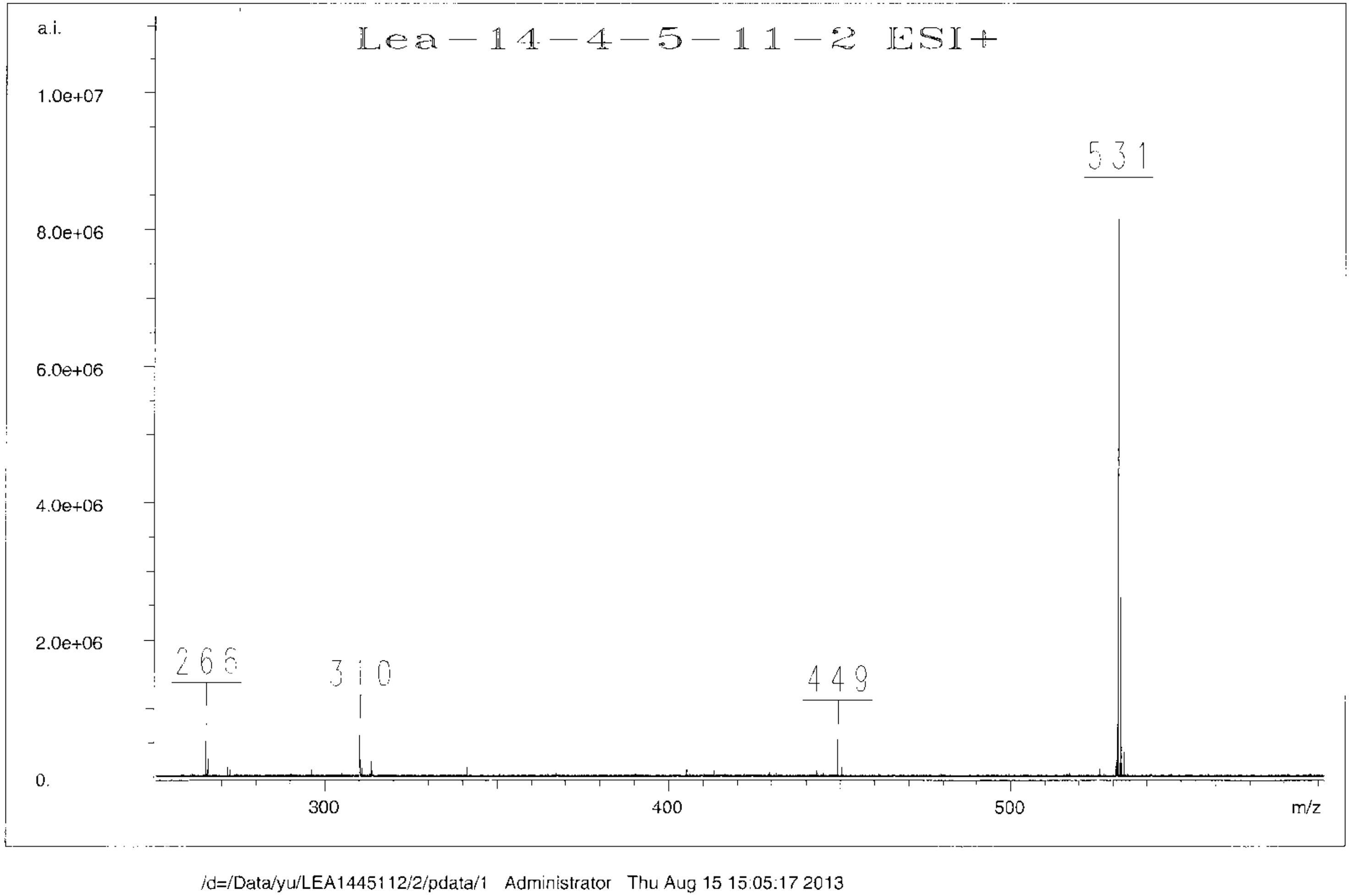

Figure S13. HRESIMS spectrum of compound **2**.

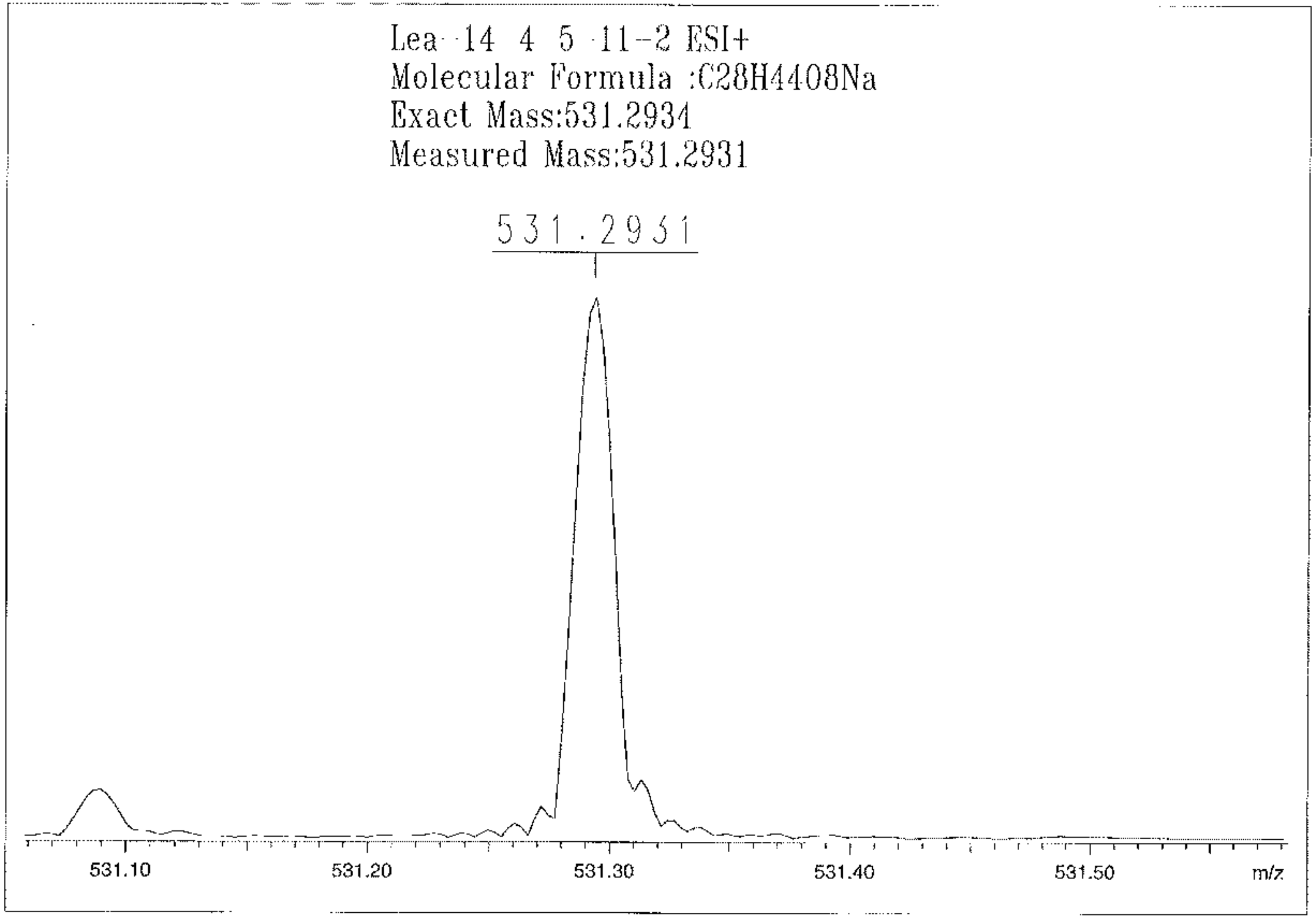

/d=/Data/yu/LEA1445112/1/pdata/1 Administrator Thu Aug 15 15:07:04 2013
